# Molecular evaluation of *Toxocara* species in stray cats using loop‐mediated isothermal amplification (lamp) technique as a rapid, sensitive and simple screening assay

**DOI:** 10.1002/vms3.431

**Published:** 2021-01-25

**Authors:** Hamid Azimian, Hamidreza Shokrani, Shirzad Fallahi

**Affiliations:** ^1^ Department of Pathobiology Faculty of Veterinary Medicine Lorestan University Khorramabad Iran; ^2^ Hepatitis Research Center Lorestan University of Medical Science Khorramabad Iran; ^3^ Department of Medical Parasitology and Mycology Faculty of Medicine Lorestan University of Medical Sciences Khorramabad Iran

**Keywords:** Iran, LAMP technique, stray cats, *Toxocara* species

## Abstract

*Toxocara* species are parasitic nematodes of dogs and cats with a worldwide distribution. The adult worm lives in the intestine, and horizontal transmission of the infection occurs through eating paratenic host or embryonated eggs. This study aimed to estimate the molecular prevalence of *Toxocara* species in stray cats using the loop‐mediated isothermal amplification (LAMP) technique. A total of 95 stool samples were randomly collected from stray cats in Khorramabad city in western Iran. Microscopic examination was performed after the separation and extraction of supernatants. The LAMP reaction was performed using the internal transcribed spacer 2 (ITS2) gene primers of *Toxocara* species and the appropriate master mix. The overall prevalence of *Toxocara* spp. in stray cats was 20% (19/95, CI 95%: 0.2 ± 0.08) by parasitological and molecular assessments. The microscopic examination of stool samples revealed that 19 samples were positive for *Toxocara*. The same 19 positive samples were also positive by the LAMP technique. Interestingly, based on the results of the LAMP assay, out of 95 studied samples, 18 (18.94%; CI 95%: 0.19 ± 0.08) specimens were *Toxocara canis*, while only 1 (1.05%; CI 95%: 0.005 ± 0.01) sample was diagnosed as *Toxocara cati*. The relatively high prevalence of *Toxocara* species in the studied cats shows the role of this species in spreading the parasite and the role of the cats in transmitting this zoonotic parasite. Preventive measures including the control of stray cat's population by castration and protection of public gardens where children play are recommended. The easy, highly sensitive and specific LAMP method is proposed for the differential detection of *Toxocara* species in animals and humans.

## Graphical abstract

*Toxocara* species are parasitic nematodes of dogs and cats with a worldwide distribution. *Toxocara* is a globally soil‐transmitted nematode and human infection has been reported in almost all regions of the world, especially those with tropical weather and abundance of dogs and cats. This parasite is spread not only in developing countries but also in all developed countries so that in some of these countries it is the most common human helminthic infection. Human infections are more commonly reported in children, especially children of low age. These nematodes in humans cause Visceral *larva migrans* (VLM) syndrome, which is accompanied by different clinical symptoms, sometimes lead to respiratory problems and even death. *Toxocara* infection can be detected in dogs and cats using history and based on clinical signs. Finding the brown eggs with thick and hollow walls in the fecal specimen determine the diagnosis. The wet mount (direct smear) preparation from the stool specimen solely is not an appropriate method for finding eggs while accompanied by the concentration methods such as flotation assay to make a more accurate diagnosis. The use of new diagnostic methods, such as molecular assays, to determine the rate of infection in dogs and cats as reservoirs of *Toxocara*, is applicable and can be compared to the results of other tests, including routine stool examination. The loop‐mediated isothermal amplification (LAMP) technique is a simple high‐performance method that was introduced by Notomi et al. (2000), in which the DNA replicated specifically, efficiently, and rapidly under isothermal conditions. The LAMP technique is a simple method that does not require expensive equipment such as Thermal cycler and Gel documentation systems and the reaction can be done in a hot water bath or thermal block. The present study aimed to investigate the molecular frequency of Toxocara species in stray cats in Khorramabad city, Western Iran, using the LAMP technique.

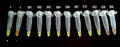

## 1 INTRODUCTION

Ascarid nematods (*Toxocara canis*, *Toxocara cati* and *Toxascaris leonina*) are parasitic roundworms of predatory mammals from families Canidae and Felidae with worldwide distribution (Eslami, 2006; Okulewicz et al., 2012). *Toxocara canis* is one of the important public health and economically important zoonotic parasitic infections in humans sharing with dogs, somewhat cats and wild canids, particularly foxes and jackals (Lee et al., 1993). The definitive hosts of *T. cati* are wild and domestic felids (Okulewicz et al., 2012). The adult worm lives in the intestine of dogs and cats, and infection of humans is caused by the ingestion of embryonated eggs or raw or undercooked liver and/or meat from paratenic hosts including cattle, sheep, ducks, pigs, lambs and chicken (Acosta et al., 2011; David & Petrie, 2006; Maruyama et al., 1994). The highest prevalence of *Toxocara* is seen in kittens and puppies of 12–24 weeks of age (O'Lorcain, 1994). Puppies may be infected by breast milk or through the umbilical cord during fetal development, kittens are also infected through breast milk (Athari, 2010; Nabavi et al., 2014; Rubinsky‐Elefant et al., 2010). The zoonotic disease resulted from these nematodes is called toxocariasis (Durant et al., 2012). *Toxocara* species do not mature in the human body, however, contamination with the second stage larvae (L2) can lead to serious complications such as high eosinophilic visceral *larvae migrans* (VLM), hepatomegaly, chronic pulmonary inflammation associated with cough and fever which can sometimes lead to breathing problems and even death (Glickman & Schantz, 1981; Kumagai et al., 2010). Toxocariasis may also cause ocular *larvae migrans* (OLM) with eye disorders as well as epilepsy and myocarditis (Alavi & Sefidgaran, 2008; Glickman & Schantz, 1981; Kumagai et al., 2010). *Toxocara* human infection has been reported in almost all regions of the world, especially those with tropical weather and abundant dog and cat populations. Approximately, 10% of the human world population are seropositive for toxocariasis (Lappin, 1993). In addition to developing countries, in some developed countries such as Germany, Hong Kong, China and Australia, toxocariasis is one of the most common human helminthic infections. Human infections are more commonly reported in children, especially children of low age (Despommier, 2003; Glickman & Schantz, 1981). Keeping dogs (especially puppies) and cats as a pet and touching and playing with these animals, geophagy by children, free entry of dogs and cats into farmland and public parks, and non‐compliance with sanitation in eating non‐washed vegetables are among the most important risk factors associated with toxocariasis (Rubinsky‐Elefant et al., 2010).

*Toxocara* infection can be detected in dogs and cats using epidemiological and clinical elements and coproscopy. The wet mount (direct smear) preparation from the stool specimen solely has low sensitivity for finding eggs while accompanied by the concentration methods such as flotation assay make a more accurate diagnosis (Dryden, 1996). The sensitivity and specificity of the floatation method for detecting *Toxocara* eggs were estimated to 51% and 100%, respectively (Overgaauw, 1997). The use of new diagnostic methods, such as molecular assays, to estimate the prevalence of infection in dogs and cats as reservoirs of *Toxocara*, is applicable and can be compared to the results of other tests, including routine stool examination. The loop‐mediated isothermal amplification (LAMP) technique is a simple high‐performance method that was developed by Notomi et al. (2000), in which the DNA replicated specifically, efficiently, and rapidly under isothermal conditions (Fallahi et al., 2014; Notomi et al., 2000). In this method, four specific designed primers (two internal and two external primers) that identify six specific regions among the target DNA are used (Kheirandish et al., 2020; Mirahmadi et al., 2020). The target region is replicated during the comet process, forming a series of the loop at a temperature of 60°C–65°C by using a thermoresistant DNA polymerase enzyme (*Bst DNA polymerase*). The LAMP technique is a simple method that does not require expensive equipment such as thermal cycler and gel documentation systems and the reaction can be done in a hot water bath or thermal block (Fallahi et al., 2015, 2018; Ghodrati et al., 2017; Ghodsian et al., 2019; Hanifehpour et al., 2019; Valian et al., 2020). The results can be simply evaluated by visual examination of the tubes and the colour change of SybrGreen I, an intercalating dye that specifically binds to the double‐stranded DNA in reaction tubes from orange to green fluorescence under daylight and UV light (Arab‐Mazar et al., 2019; Fallahi et al., 2020; Kaneko et al., 2007; Mori et al., 2007; Nagamine et al., 2001).

The high population of stray cats, as one of the definitive hosts of *Toxocara,* in Khorramabad city, Western Iran, the low number of studies conducted in this area, and the high resistance of *Toxocara* eggs, as a major source of environmental contamination, emphasizes the need for research on the prevalence of *Toxocara* in stray cats.

## 2 MATERIALS AND METHODS

### 2.1 Samples collection

During the 3‐month period (July–September 2018), a total of 95 stool samples were randomly collected from stray cats in Khorramabad city, Lorestan province in west of Iran. Stool samples were immediately transferred to the Parasitology Laboratory Faculty of Medicine, Lorestan University of Medical Sciences, Iran, and divided into two parts. One part of each specimen was prepared for parasitological examinations, and the other part was kept at −20°C for DNA extraction and subsequent molecular evaluation.

### 2.2 Parasitological examinations

The stool specimens were poured into a tube containing isotonic saline (0.85% NaCl solution) and vortexed for 5 min. The homogenized samples were examined by microscopic observation (Zeiss, Germany, ×100 and ×400 magnification) of direct smears using isotonic saline, and floatation with a saturated chlorine solution. To separate the debris, the suspensions were passed through two layers of wet tampons. Thereafter, the samples were centrifuged at 3,000*g* for 3 min, the supernatant was discarded and the saturated chlorine solution was spilled into the tube and centrifuged at 2,000*g* for 5 min. Then, 300 μl of the supernatant solution was removed and poured into a 1.5‐ml microtube. *Toxocara* eggs were identified by microscopic observation of supernatant under ×100 and ×400 magnification.

To rupture *Toxocara* eggshell and allow DNA extraction, sonication and freezing‐thawing methods were used. First, the sonication cycle of the sonicator machine was set to 0.5 cycles, and the voltage to 70 V. The microtubes containing the specimens were placed inside the ice and subsequently, each sample was sonicated for 20 s (5 times every 4 s). Subsequently, the samples were placed five times every 2 min inside the liquid nitrogen then water bath at 95°C.

### 2.3 Molecular examination

DNA extraction was performed using a DNA extraction kit (Stool DNA Isolation mini Kit, Yekta Tajhiz Azma Co. Iran) based on the protocol of the manufacturer. The LAMP technique was performed targeting the highly conserved ITS2 gene of *Toxocara* spp. with a set of 4 primers (Table 1; Macuhova et al., 2010). LAMP reaction was carried out in a final volume of 25 μl (Fallahi et al., 2014). Since the loop primers were not designed for the ITS2 gene of both *T. canis* and *T. cati,* in the LAMP reaction, double‐distilled water replaced loop primers. The reaction tubes were placed in a water bath at 65°C for 1 hr. To the visual assessment of the LAMP amplicons, 3 µl of SYBR Green I (Invitrogen, Thermo Fisher Scientific Invitrogen lot, Carlsbad, California, United States) diluted in dimethyl sulfoxide (DMSO; Sigma‐Aldrich, St. Louis, Missouri, United States), was added to each tube and observed under daylight and UV light. Furthermore, the gel electrophoresis was performed on the LAMP products in a 1.5% agarose gel stained with DNA safe stain (1 µg/ml; Sinaclon Co., Tehran, Iran) and visualized under UV light. The assay repeatability was evaluated by performing LAMP reactions with duplicates for all stool samples. Genomic DNA from *Toxocara* spp. standard strain and doubled distilled water were included in each LAMP reaction as positive and negative controls, respectively.

**TABLE 1:** Nucleotide sequence of primers targeting the ITS2 gene of *Toxocara* spp. used in the LAMP reaction (Macuhova et al., 2010)

Target *Toxocara* species	Primer set	Primer sequence (5'–3')
*Toxocara cati*	Tcati‐F3 Tcati‐B3 Tcati‐FIP Tcati‐BIP	ccacgtaccttgccaagac gcgcattccttcttcaagca ggaacacatacgccaatggccatgcacaagaaatcgctgtcg acgatatggcctccagcaagccgatgacgttacctccaacc
*Toxocara canis*	Tcan‐F3 Tcan‐B3 Tcan‐FIP Tcan‐BIP	tgtgattaacgcgcaaggt ctggaggccgtatcgtga ccttggcaaggtacgctgtacatgtggtgcattcggtgag tcgcacaagaaatggctgtcgtagcaacgcaacatacactca

## 3 RESULTS

### 3.1 Parasitological examinations

**FIGURE 1 a & b:**
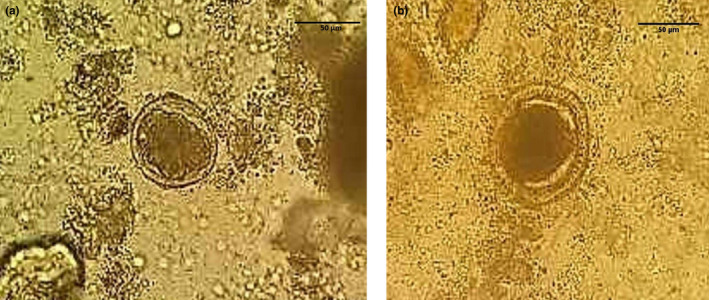
*Toxocara* spp. eggs in cat's stool

Based on parasitological examinations of the stool specimens by direct smears (wet mount) and floatation with a chlorine solution, the prevalence of *Toxocara* spp. was 20% (19/95; CI 95%; 0.2 ± 0.08) among stray cats of Khorramabad in western Iran (Figure 1). The number of positive samples detected by the flotation and wet mount direct smears methods were 12 and 7, respectively (data not shown).

### 3.2 Molecular examination

**FIGURE 2:**
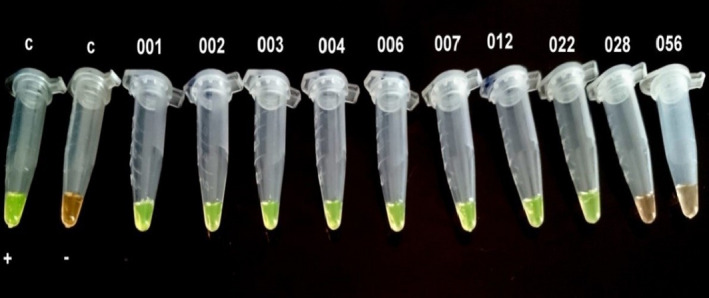
Tubes after LAMP reaction performed on stool samples from stray cats using SYBR Green I DNA stain under UV light. C+; Positive control, C−; Negative control, microtubes 001, 002, 003, 004, 006, 007, 012, and 022; represent the positive LAMP results for *Toxocara canis* in the DNA samples from stray cat's feces

**FIGURE 3:**
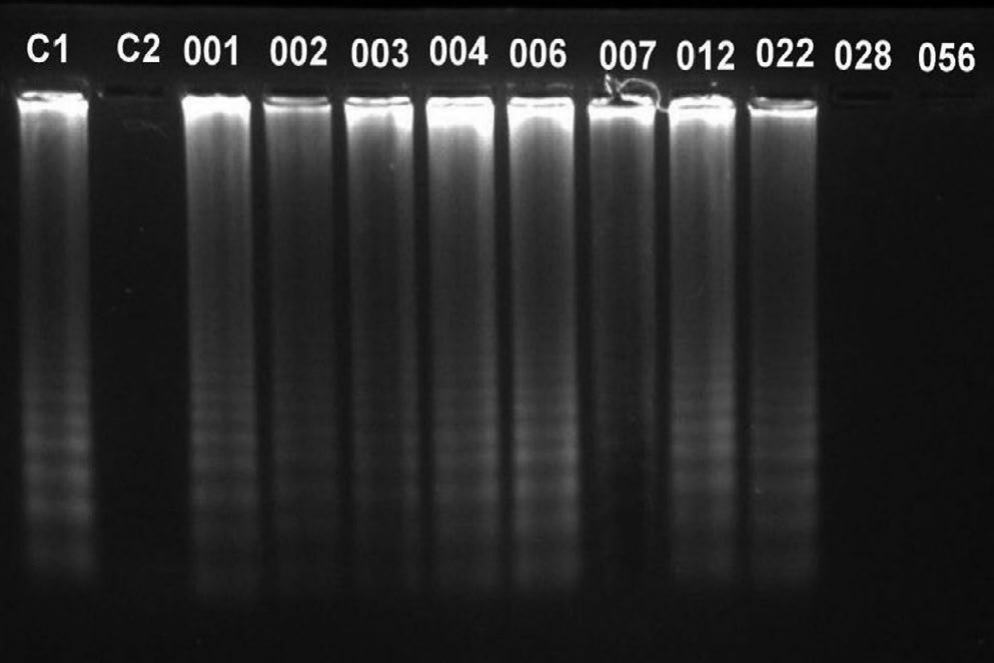
Evaluation of LAMP products by electrophoresis in 1.5% agarose gel stained by SYBR Safe DNA gel stain. C1; Positive control, C2; Negative control, lanes 001, 002, 003, 004, 006, 007, 012, and 022; represent the positive LAMP results for *Toxocara canis* in the DNA samples from stray cat's feces

**FIGURE 4:**
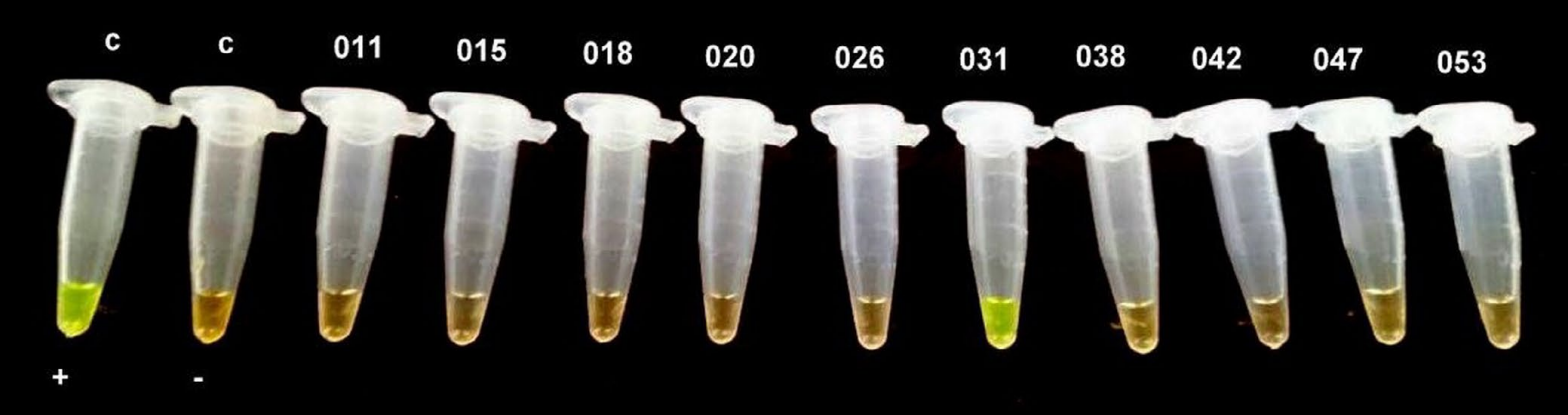
Tubes after LAMP reaction performed on stool samples from stray cats using SYBR Green I DNA stain under UV light. C+; Positive control, C‐; Negative control, microtube 031; represent the only positive LAMP result for *Toxocara cati* in the DNA sample from stray cat's feces

**FIGURE 5:**
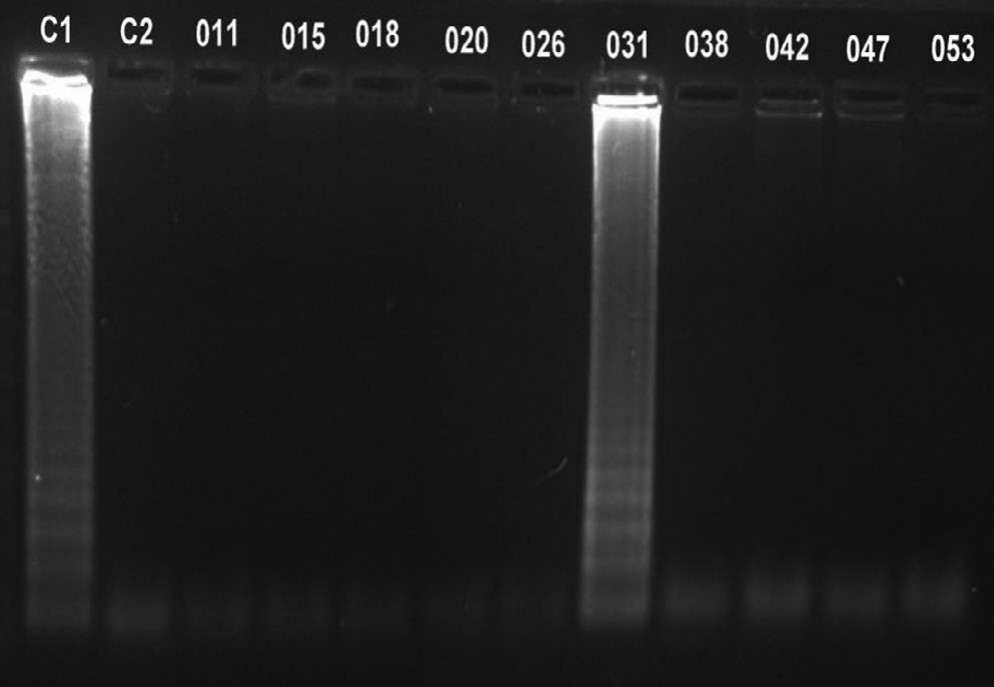
Evaluation of LAMP products by electrophoresis in 1.5% agarose gel stained by SYBR Safe DNA gel stain. C1; Positive control, C2; Negative control, lane 031; represent the only positive LAMP result for *Toxocara cati* in the DNA samples from stray cat's feces

Out of 95 studied samples, 18 (18.94%; CI 95%: 0.19 ± 0.08) were infected by *T. canis* (Figures 2 and 3) and 1 sample (1.05%; CI 95%: 0.005 ± 0.01) was infected by *T. cati* (Figures 4 and 5).

## 4 DISCUSSION

Considering the effect of *Toxocara* spp. on human health including VLM and OLM syndromes, hepatomegaly, pulmonary involvement, myocarditis, epilepsy and even death, as well as the high pathogenicity in cats and dogs, specially puppies, detection of parasites in these animals, as definitive hosts of the parasites is of great importance (Kleine et al., 2017; Kumagai et al., 2010). Based on the results of the present study, 20% (19/95; CI 95%: 0.2 ± 0.08) of the studied cats were infected by *Toxocara* spp., among them, 18 animals (18.94%; CI 95%: 0.19 ± 0.08), were infected by *T. canis* while only one animal (1.05%; CI 95%: 0.005 ± 0.01) was infected by *T. cati*. These results indicate the relatively high prevalence of *Toxocara* species, especially *T. canis*, in stray cats in Khorramabad in western Iran, which shows the potential role of these animals as important reservoirs for other animals and humans, especially children. The results of the present study are consistent with those carried out in different regions of Iran on cats in terms of a relatively high *Toxocara* prevalence (Arbabi & Hooshyar, 2009; Changizi et al., 2007; Khademvatan et al., 2013; Sharif et al., 2007; Torkan et al., 2017). However, contrary to other studies, the dominant species in the current study was *T*. *canis*. Such result could be due to the high prevalence of stray dogs in Khorramabad city in western Iran, high contamination of the environment, and possibly the transmission of infection to stray cats. There have been two reports from Malaysia of a ‘variant’ ascaridoid of cats assigned to *T. canis* (Lee et al., 1993; Rohde, 1962). Lee et al. (1993) reported that of the 55 examined cats from Kuala Lumpur suburbs, Malaysia, 15 were infected by *Toxocara* spp. of these, 12 were infected by *T*. *cati* and the others by *T*. *canis*. Likewise, Parsons and colleagues (1988) reported a disseminated granulomatous disease within the kidneys, ventricles, liver, lungs, spleen, diaphragm and intestinal serosa of cats caused by *T. canis* larvae. In a study conducted by Zibaei et al. (2010) using the sucrose flotation method, 63.3% of 285 soil samples from public parks in Khorramabad, Iran were contaminated by *Toxocara* eggs (Zibaei et al., 2010). The result of this study showed the high environmental contamination caused by dogs and cats in Khorramabad city. In the present study, there was no discrepancy between the results of microscopy and the LAMP technique for *Toxocara* detection, and a perfect concordance was obtained between the two methods. However, the morphological recognition of the *Toxocara* species is difficult by microscopic examination, while the LAMP method can help in differential diagnosis of the species. Similar to the results of the present study, no difference was found between microscopic method and 2qPCR technique, a specific and rapid Duplex quantitative real‐time PCR on the ITS‐2 gene of *Toxocara* that was used by Durant et al. (2012) to detect *T. cati* and *T. canis* in soil and faeces samples. Ozlati et al. (2016) in Tabriz city in north‐west Iran, studied 180 samples of suspected contaminated soil using microscopy, PCR, and LAMP techniques. Their results showed that 57, 14 and 77 samples were positive for *Toxocara* by microscopy, PCR and LAMP methods, respectively. Among them 49 samples (27.2%) were contaminated by *T*. *cati* and 28 (15.5%) by *T. canis*. The results of Ozlati and colleagues (2016) showed a higher sensitivity of LAMP (detection limit 1–3 eggs/200 g soil) than PCR (detection limit > 3 eggs/200 g soil) and microscopy techniques.

The regional difference of *Toxocara* prevalence could be explained by different factors including the density and numbers of dogs, cats and rodents in the study area, the public health conditions, the season in which the study was carried out, and the diagnostic technique applied for parasite detection (Badparva et al., 2009; Torkan et al., 2017). The development of parasite eggs in the environment depends on the temperature and humidity of the environment. Higher temperature and humidity accelerate egg development and as the definitive hosts, dogs and cats are more likely to be infected. As paratenic hosts of *Toxocara* spp., rodent populations play an important role in the epidemiology of this parasitic infection. Studies in different regions of the world showed a high prevalence of *Toxocara* spp. in the main hosts and the environment. Macuhova et al. (2010) in Japan, used the LAMP and PCR techniques targeting the ITS‐2 gene of *Toxocara* to screen the *T. cati* and *T. canis* in five sand samples, their results showed that four out of five samples were contaminated by *T. cati*, and none by *T. canis*. The LAMP technique, with its robust *Bst DNA polymerase* enzyme and numerous primers, has very high sensitivity and specificity in the diagnosis of various infections, including parasites (Arab‐Mazar et al., 2016). After culture and testing faeces of 58 cats, in Spain, 55.2% of them were diagnosed infected by *T. cati* (Calvete et al., 1998). In a study on stray cats in Mizoram, India, 85.2% of the cats were infected by various parasites, and *T. cati* was the second most prevalent parasite after *Taenia taeniaformis* (Borthakur & Murkharjee, 2011). In Hussam and Aredhi (2015) investigated 90 fecal samples of cats in Iraq, of which 43 (47.8%) were infected by intestinal parasites that the prevalence of *T. cati* was 25.6%.

Identification of *Toxocara* species in dogs and cats as definitive hosts of the parasite is important because they are the main source of human infections especially for children, as well as the pathogenicity of parasites to their definite hosts especially dogs is high and can even be fatal in puppies. The relatively high prevalence of *Toxocara* species in the studied cats shows the potential role of these animals in spreading the disease and the risk of transmission to humans. Preventive measures including the control of stray cat's population by castration, protection of public gardens where children play, periodic screening, and treatment of dogs and cats are recommended. Due to its sensitivity, specificity, fastness and low cost, the LAMP technique is proposed for the differential detection of *Toxocara* species in animals and humans.

## CONFLICT OF INTEREST

The authors declare that they have no conflict of interest.

## AUTHOR CONTRIBUTION

**Hamid Azimian:** Investigation; Project administration. **Hamidreza Shokrani:** Conceptualization; Data curation; Funding acquisition; Validation; Writing‐review & editing. **Shirzad Fallahi:** Conceptualization; Data curation; Investigation; Methodology; Project administration; Supervision; Validation; Visualization; Writing‐original draft.

## ETHICAL STATEMENTS

The authors confirm that the ethical policies of the journal, as noted on the journal's author guidelines page, have been adhered to. The study was approved by the ethical committee of the Lorestan University, Khorramabad, Lorestan Province, West of Iran.

### Peer Review

The peer review history for this article is available at https://publons.com/publon/10.1002/vms3.431.
